# Short-term effects of ambient fine particulate matter pollution on hospital visits for chronic obstructive pulmonary disease in Beijing, China

**DOI:** 10.1186/s12940-018-0369-y

**Published:** 2018-02-27

**Authors:** Yaohua Tian, Xiao Xiang, Juan Juan, Jing Song, Yaying Cao, Chao Huang, Man Li, Yonghua Hu

**Affiliations:** Department of Epidemiology and Biostatistics, School of Public Health, Peking University, No.38 Xueyuan Road, Beijing, 100191 China

**Keywords:** COPD, PM_2.5_, Air pollution, Outpatient visit, Inpatient visit

## Abstract

**Background:**

Little is known about the effect of ambient fine particulate matter (PM_2.5_) on chronic obstructive pulmonary disease (COPD) in China. The objective of this study was to explore the short-term effects of PM_2.5_ on outpatient and inpatient visits for COPD in Beijing, China.

**Methods:**

A total of 3,503,313 outpatient visits and 126,982 inpatient visits for COPD between January 1, 2010, and June 30, 2012, were identified from the Beijing Medical Claim Data for Employees. A generalized additive Poisson model was applied to estimate the percentage change with 95% confidence interval (CI) in hospital visits for COPD in relation to an interquartile range (IQR) (90.8 μg/m^3^) increase in PM_2.5_ concentrations.

**Results:**

Short-term exposure to PM_2.5_ was significantly associated with increased use of COPD-related health services. There were clear exposure–response associations of PM_2.5_ with COPD outpatient and inpatient visits. An IQR increase in the concurrent day PM_2.5_ concentrations was significantly associated with a 2.38% (95% CI, 2.22%–2.53%) and 6.03% (95% CI, 5.19%–6.87%) increase in daily outpatient visits and inpatient visits, respectively. Elderly people were more sensitive to the adverse effects. The estimated risk was higher during the warm season compared to the cool season.

**Conclusions:**

Short-term exposure to PM_2.5_ was associated with increased risk of hospital visits for COPD. Our findings contributed to the limited evidence concerning the effects of ambient PM_2.5_ on COPD morbidity in developing countries.

**Electronic supplementary material:**

The online version of this article (10.1186/s12940-018-0369-y) contains supplementary material, which is available to authorized users.

## Background

Chronic obstructive pulmonary disease (COPD) is a major public health problem that affects more than 300 million people and accounts for an estimated 3.2 million deaths worldwide in 2015 [1, 2]. In China, the number of COPD cases increased from 32.4 million in 1990 to 54.8 million in 2013 [3]. Research has provided compelling evidence linking ambient air pollution to COPD risk [4–8]. Ambient fine particulate matter (PM_2.5_, particulate matter ≤2.5 μm in aerodynamic diameter) has been a leading cause of global burden of disease, accounting for an estimated 4.2 million deaths and 103.1 million disability-adjusted life-years in 2015 [9]. A recent meta-analysis that summarized the results from 12 studies using either admission or mortality data indicated that short-term exposure to PM_2.5_ was significantly associated with increased risk of COPD [10]. However, none of these studies were conducted in developing countries where PM_2.5_ pollution is generally more severe [11], possibly because of the limited availability of PM_2.5_ monitoring data. For example, in China, data on PM_2.5_ level in major Chinese cities were first announced in 2013.

Short-term increase in PM_2.5_ concentration has been linked to excess daily emergency room visits, hospital admissions and mortality due to COPD. However, few studies have been conducted to evaluate the effect of PM_2.5_ on daily office-based physician visits, mainly because regular outpatient visits in Western countries are scheduled by appointment [12]. In the U.S., in 2000, 8 million visits for COPD were made to physician offices, while there were only 1.5 million emergency department visits, 726,000 hospitalizations and 119,000 deaths for COPD [13, 14]. Therefore, outpatient physician visit could reflect air pollution-related effects in a broader segment of population with its greater coverage [15–17].

China, the largest developing country, has the highest ambient PM_2.5_ levels worldwide [18]. A general practitioner-based referral system is not available in China [19]. Regular outpatient visits are unscheduled and are on a first-come first-served basis. Hospital outpatient and emergency department visits were estimated to account for > 95% of total hospital visits in 2014 [17]. Outpatient visit has been used as an important morbidity measure in assessing air pollution-related health effects in China [15, 17, 20, 21]. The objective of this study was to explore the short-term effects of PM_2.5_ on hospital visits for COPD in Beijing, China.

## Methods

### Data collection

Daily counts of hospital visits for COPD were obtained from Beijing Medical Claim Data for Employees. The database records medical claim data for all working or retired employees who are covered by basic medical insurance in Beijing. In order to be reimbursed, a claim for billable medical service must be submitted on a standardized electronic form, which includes data elements such as gender, birthday, the date of hospital visit, discharge diagnosis in Chinese and corresponding International Classification of Diseases, 10th Revision (ICD-10) code, and reimbursement information. Daily outpatient and inpatient visits with a primary diagnosis of COPD (ICD-10 codes J40–J44) between January 1, 2010, and June 30, 2012 (a total of 912 days) were extracted from the database. The outpatient visit was defined as a patient visit to a physician’s office, clinic, or hospital outpatient department [22]. Patients aged < 18 years were too few and thus were excluded from this analysis.

Data on hourly PM_2.5_ concentrations were collected from the reports published by the U.S. embassy, which established an ambient air quality monitoring station on the rooftop of embassy building located in Chaoyang district, Beijing. The location of the monitoring station was shown in the Additional file 1: Figire S1. The PM_2.5_ levels obtained from the monitor have been demonstrated to exhibit approximately the same trend as city-wide PM_2.5_ levels [23]. To reduce exposure misclassification, the maximum distance between the monitor and hospital visits considered was approximately 40 km [24, 25]. Approximately 79.2% of Beijing’s total population lived within a 40-km radius of the monitor. All areas of high population density (> 5000 people/km^2^), 97.8% (44/45) of the tertiary hospitals and 79.3% (69/87) of the secondary hospitals in Beijing located within a 40-km radius of the monitor [26]. Previous studies have indicated that the monitoring data could be used as a proxy for population exposure among individuals living < 40 km from the monitor [26–28]. The reliability of PM_2.5_ measurements has been validated in previous studies [24, 26]. We used daily (24-h) mean concentrations of PM_2.5_ as a proxy for population exposure level. We also obtained meteorological data on temperature (°C) and relative humidity (%) during the study period from the Chinese Meteorological Bureau.

### Statistical analysis

We examined the association between PM_2.5_ and hospital visits for COPD using a generalized additive Poisson model.

Log[E(Y_t_)] = α + βPM_2.5_ + public holiday + day of week + *ps*(calendar time, 6 per year) + *ps*(Temperature, 3) + *ps*(Relative humidity, 3).

Where, E(Y_t_) is the expected daily count of hospital visits for COPD on day t; *ps*() indicates penalized spline function; public holiday and the day of week were adjusted for as categorical variables; β represents log-relative risk of COPD morbidity in relation to unit increase in PM_2.5_ concentrations. We applied the distributed lag non-linear models with three degrees of freedom (*df*) in the penalized splines and a maximum lag of 3 days to control the effects of weather conditions [29]. The *df* values for calendar time, temperature, and relative humidity used in this analysis were in line with previous studies [17, 21]. We also assessed the robustness of the results in terms of the *df* values for time trend (4–8 per year), temperature (2–6) and relative humidity (2–6).

Because the assumption of the linearity between PM_2.5_ level and hospital visits may not be justified, we explored the non-linear exposure-response association using a penalized cubic regression spline of PM_2.5_ concentration with 3 degrees of freedom. To explore the temporal association between COPD hospital visits and PM_2.5_, we fitted the models with single-day lag from the current day (lag 0) up to previous 3 days (lag 3). We also estimated associations with 2-day (lag 0–1), 3-day (lag 0–2), and 4-day (lag 0–3) moving average concentrations. We further explored potential effect modification of COPD risk by sex, age (18–64 years and ≥65 years), and season (warm: April to September; cool: October to March) using concurrent day PM_2.5_ concentration. A penalized spline function of calendar time on warm or cool season was used to accommodate the long-term trend in hospital visits for COPD [30, 31]. The statistical significance of subgroup differences were tested using the Z-test [32].

The results were presented as the percentage changes and 95% confidence intervals (CIs) in daily COPD hospital visits associated with per interquartile (IQR) (90.8 μg/m^3^) increase in PM_2.5_ concentration. All analyses were conducted in R Programming Language (V.3.2.2, R Development Core Team) using the “*mgcv*” and “*nlme*” packages.

## Results

A summary of basic descriptive information is provided in Table 1. A total of 3,503,313 outpatient visits and 126,982 inpatient visits between January 1, 2010, and June 30, 2012, formed the basis of this study. The mean ages (SD) for outpatient and inpatient visits were 64.3 (12.9) and 71.6 (12.2) years, respectively. For outpatient visits, there were 53.6% male patients and 51.1% elderly patients (aged ≥65 years). For inpatient visits, there were 60.1% male patients and 74.9% elderly patients. Table 2 shows the distribution of daily hospital visits for COPD, PM_2.5_ concentration and meteorological variables in Beijing. Over the study period, the daily mean (SD) counts of outpatient and inpatient visits were 3854 (3199) and 44 (31), respectively. The daily mean (SD) PM_2.5_ concentrations was 99.5 (75.3) μg/m^3^. Of the 912 days, only 414 (45.4%) days of daily PM_2.5_ concentrations achieved the target of the Chinese Ambient Air Quality Standards Grade II standards (≤ 75 μg/m^3^), and 124 (13.6%) days achieved the target of WHO Air Quality Guidelines (≤ 25 μg/m^3^). A scatter plot on PM_2.5_ concentration and counts of outpatient and inpatient visits was shown in the Additional file 1: Figure S2.

**Table 1:** Demographic characteristics of chronic obstructive pulmonary disease (COPD) hospital visits between January 1, 2010, and June 30, 2012, in Beijing, China

Variable	No.
Outpatient visits	3,503,313
Sex	
Male (%)	1,878,395 (53.6)
Female (%)	1,624,918 (46.4)
Age (year) (mean ± SD)	64.3 ± 12.9
< 65 (%)	1,714,404 (48.9)
≥ 65 (%)	1,788,909 (51.1)
Inpatient visits	126,982
Sex	
Male (%)	76,357 (60.1)
Female (%)	50,625 (39.9)
Age (year) (mean ± SD)	71.6 ± 12.2
< 65 (%)	31,870 (25.1)
≥ 65 (%)	95,112 (74.9)

**Table 2:** Summary statistics for daily count of chronic obstructive pulmonary disease (COPD) hospital visits, daily fine particulate matter (PM_2.5_) concentrations and weather conditions between January 1, 2010, and June 30, 2012, in Beijing, China

			Percentile				
Variable	Mean ± SD	Minimum	25th	50th	75th	Maximum	IQR
Outpatient visits	3854 ± 3199	15	1326	3427	5585	16,920	4259
Inpatient visits	44 ± 31	1	93	131	176	409	83
PM_2.5_ (μg/m^3^)	99.5 ± 75.3	7.2	42.5	82.8	133.3	492.8	90.8
Temperature(°C)	12.6 ± 11.6	−12.5	1.5	14.1	23.8	34.5	22.3
Relative humidity (%)	48.6 ± 20.3	9	30	48	66	92	36

*IQR* Interquartile range, *SD* Standard deviation

**Fig. 1:**
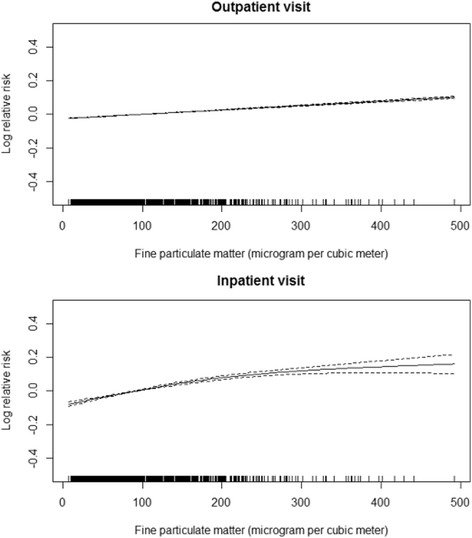
The concentration-response curves of fine particulate matter (PM_2.5_) concentrations (degree of freedom = 3) and outpatient visits and inpatient visit for chronic obstructive pulmonary disease (COPD) between January 1, 2010, and June 30, 2012, in Beijing, China. Note: The X-axis is the concurrent day PM_2.5_ concentrations (μg/m^3^), Y-axis is the predicted log (relative risk (RR)), is shown by the solid line, and the dotted lines represent the 95% CI

There were clear exposure–response associations of PM_2.5_ with COPD outpatient and inpatient visits (Fig. 1). Table 3 shows percentage changes in hospital visits associated with an IQR increase in PM_2.5_ concentration for different lag structures. We observed significant association between PM_2.5_ and hospital visits after adjustment for calendar time, day of the week, public holiday, and weather conditions. An IQR increase in PM_2.5_ concentration on the same day corresponded to a 2.38% (95% CI, 2.22%–2.53%) and 6.03% (95% CI, 5.19%–6.87%) increase in outpatient visits and inpatient visits, respectively. For easy comparisons with other studies under discussion, we have provided a table representing the percentage changes in daily COPD hospital visits associated with per 10 μg/m^3^ increase in PM_2.5_ concentrations in the Additional file 1: Table S1.

**Table 3:** Percentage changes with 95% CIs in outpatient and inpatient visits for chronic obstructive pulmonary disease (COPD) associated with an interquartile range increases in fine particulate matter (PM_2.5_) concentration (90.8 μg/m^3^) for different lag structures

Hospital service	Lag days	Percentage change	95% CI	*P*
Outpatient visits	Lag 0 days	2.38	2.22–2.53	<2e-16
	Lag 1 days	0.19	0.06–0.33	0.00485
	Lag 2 days	0.87	0.75–1.00	<2e-16
	Lag 3 days	1.57	1.44–1.69	<2e-16
	Lag 0–1 days	1.62	1.44–1.79	<2e-16
	Lag 0–2 days	1.81	1.64–2.00	<2e-16
	Lag 0–3 days	2.43	2.23–2.62	<2e-16
Inpatient visits	Lag 0 days	6.03	5.19–6.87	<2e-16
	Lag 1 days	2.41	1.69–3.14	4.46e-11
	Lag 2 days	1.29	0.63–1.96	0.000119
	Lag 3 days	0.21	−0.44-0.86	0.527
	Lag 0–1 days	5.61	4.68–6.54	<2e-16
	Lag 0–2 days	5.06	4.09–6.05	<2e-16
	Lag 0–3 days	4.33	3.31–5.36	<2e-16

Table 4 shows the estimates of season-, sex- and age-specific effects for PM_2.5_. In the season-specific analysis, stronger associations were observed in the warm season for both outpatient and inpatient visits. Stronger effects were also observed in the elderly people and females. In the sensitivity analyses by changing the degrees of freedom for calendar time (4–8), temperature (2–6) and relative humidity (2–6), the results remained consistent, indicating that the association between PM_2.5_ and COPD morbidity obtained from the main models was robust (Table 5).

**Table 4:** Percentage changes with 95% CIs in outpatient and inpatient visits for chronic obstructive pulmonary disease (COPD) associated with an interquartile range increases in fine particulate matter (PM_2.5_) concentration (90.8 μg/m^3^) by season, sex and age^a^

	Percentage change	95% CI	*P*-value
Outpatient visits			
Season^b^			< 0.001
Cool	2.19	1.99–2.40	
Warm	2.80	2.51–3.09	
Sex			< 0.001
Male	2.10	1.88–2.31	
Female	2.70	2.47–2.93	
Age (year)			< 0.001
< 65	1.41	1.18–1.63	
≥ 65	3.30	3.08–3.53	
Inpatient visits			
Season^b^			< 0.001
Cool	2.96	1.91–4.01	
Warm	8.35	6.76–9.98	
Sex			0.982
Male	6.03	4.94–7.12	
Female	6.01	4.69–7.34	
Age (year)			0.005
< 65	4.21	2.61–5.82	
≥ 65	6.72	5.73–7.71	

^a^Lag 0 concentrations were used

^b^Cool season: from October to March; Warm season: from April to September

**Table 5:** Percentage changes with 95% CIs in outpatient and inpatient visits for chronic obstructive pulmonary disease (COPD) associated with an interquartile range increases in fine particulate matter (PM_2.5_) concentration (90.8 μg/m^3^) on the same day, by different degree of freedom (*df*) for calendar time, temperature, and relative humidity

		Outpatient visits		Inpatient visits	
Variable	*df*	Percentage change	95% CI	Percentage change	95% CI
Calendar time	4	1.26	1.11–1.41	5.30	4.47–6.14
	5	2.38	2.22–2.53	6.03	5.19–6.87
	6^a^	2.38	2.22-2.53	6.03	5.19–6.87
	7	2.38	2.22–2.53	6.03	5.19–6.87
	8	2.37	2.21–2.52	6.01	5.17–6.86
Temperature	2	2.38	2.22–2.53	6.03	5.19–6.87
	3^a^	2.38	2.22-2.53	6.03	5.19–6.87
	4	2.88	2.72–3.04	6.42	5.57–7.29
	5	2.66	2.50–2.82	6.52	5.67–7.39
	6	2.33	2.17–2.49	6.41	5.55–7.27
Relative humidity	2	2.38	2.22–2.53	6.03	5.19–6.87
	3^a^	2.38	2.22-2.53	6.03	5.19–6.87
	4	2.28	2.12–2.44	5.89	5.05–6.74
	5	2.25	2.09–2.40	5.84	5.00–6.68
	6	2.25	2.09–2.41	5.87	5.03–6.72

^a^The *df* value used in this study model

## Discussion

In this city-wide time-series study, we examined the association between PM_2.5_ exposure and daily hospital visits for COPD in Beijing. PM_2.5_ was positively associated with both outpatient and inpatient visit for COPD. There were substantial differences in the effect estimates between inpatient visit and outpatient visit. To the best of our knowledge, this is the first study to examine the differences in the effects of PM_2.5_ on COPD-attributed outpatient visit and inpatient visit. We found that females and elderly were more vulnerable to the adverse effects of PM_2.5_. In addition, the risk estimates were higher during the warm season compared to the cool season.

Previously, associations of PM_2.5_ with emergency department visits or hospital admissions for COPD have been extensively examined in Western countries [4–7]. For example, in a meta-analysis of 12 time-series or case-crossover studies of PM_2.5_ and daily hospitalizations for COPD, most of which were conducted in Europe and the U.S., Li et al. [10] estimated that the excess change in COPD hospitalizations associated with a 10 μg/m^3^ increase in PM_2.5_ (lag days 0–7) was 3.1% (95% CI: 1.6%–4.6%). Furthermore, a recent meta-analysis of East Asian literature also indicated significant impacts of PM_2.5_ on COPD morbidity [33]. However, few studies in China have addressed the association of PM_2.5_ with morbidity risk. A study conducted in two public general hospitals in Jinan city demonstrated that an increase of 10 μg/m^3^ in PM_2.5_ concentration corresponded to a 1.4% (95% CI: 0.7%–2.1%) and 1.5% (95% CI: 0.4%–2.6%) increase in respiratory emergency department visits for the urban and suburban population, respectively [34]. Another study conducted in ten general hospitals in Beijing found that every 10 μg/m^3^ in PM_2.5_ concentration was significantly associated with a 1.46% (95% CI: 0.13%–2.79%) increase in the emergency department visits for acute exacerbation of COPD on the same day [35]. A common limitation of these studies was their restriction to one or several hospitals. The use of a large city-wide population-based database in this study helped ensure the representativeness and generalizability of our findings. Our findings were supported by a recent national study done in 272 cities in China that reported significant effects of PM_2.5_ on mortality [8].

To date, only a very limited number of studies have evaluated the acute effects of air pollution on outpatient visits for COPD. We observed a significant association between PM_2.5_ and outpatient visits for COPD exacerbations in Beijing. A study in Taiwan demonstrated that outpatient visits for COPD shows positive correlation with PM_2.5_ [16]. Our findings are also supported by a recent time-series study conducted in a hospital in Dongguan, China, that reported a significant effect of PM_2.5_, and that an IQR (33.61 μg/m^3^) increase in PM_2.5_ concentration at lag 0–3 day was associated with a 8.32% (95% CI, 0.85%–16.33%) increase in daily outpatient visits for COPD [21]. Similarly, a case-crossover analysis conducted in a hospital in Beijing, demonstrated that each 10 μg/m^3^ increase of PM_2.5_ concentration on the current day corresponded to 0.1% (95% CI: 0%, 0.3%) increase in daily outpatient visits for acute exacerbation of COPD [36]. As this is the first study in China to demonstrate a significant effect of PM_2.5_ on outpatient visits for COPD at the city level, future studies are needed to confirm our findings.

The effect estimates for inpatient visits appeared to differ from those for outpatient visits. Our findings are supported by a time-series analysis in Shanghai that reported significant effects of PM_2.5_ on daily emergency room visits, but not on outpatient visits [37]. Outpatient visits, emergency room visits and hospitalizations are three major types of health service utilization, but also possessed of important disparities. When the concentration of PM_2.5_ rises, patients with mild or moderate COPD exacerbations usually expect to receive treatment or fill prescription in outpatient department. Subsequently, if the condition was deteriorated or ineffective, they would then be transferred to emergency department or directly to the ward. Therefore, analyzing outpatient visit provides a more comprehensive estimate of the effect of air pollution, especially for mild and moderate exacerbations. The distinct risk estimates on outpatient visit and inpatient visit might be attributable to the difference in the severity of COPD between these two morbidity outcomes. The factors causing the variations of inpatient visit and outpatient visit can be complicating. Future studies are warranted to explore the variations in the effects estimates for different morbidity outcomes.

Exploring the potential effect modifiers is helpful to identify potentially susceptible population and to develop a more accurate targeted intervention. We found that the effects of PM_2.5_ were significantly greater in the elderly people, which are consistent with previous findings [6, 35, 38]. These evidences suggested that elderly people, especially those presenting with COPD, should limit outdoor activity or wear a face mask outdoors to reduce personal exposure when PM_2.5_ pollution is severe. This is in line with the protective measures recommended by government [39]. The gender subgroup analysis suggested that females were more susceptible to PM_2.5_ exposure among outpatient visits, but not among inpatient visits. The gender-specific acute effects of PM_2.5_ exposure on COPD morbidity were inconsistent in prior studies [6, 10, 35]. In this study, stronger PM_2.5_ effects were observed in the warm season. Our findings were consistent with several previous studies [40–42], but in conflict with others indicating non-significant seasonal pattern or even greater adverse effects in the cool season [43]. During the warm season, Beijing residents are more likely to go outdoors and open windows; thus, monitored PM_2.5_ concentrations may be closer to personal exposure. In addition, seasonal differences in air pollutants may also affect the effect estimates. The seasonal variation in effect estimates need to be further investigated.

This study was subject to several limitations. First, the use of PM_2.5_ concentrations derived entirely from a fixed-site monitoring station as a proxy for personal exposure is expected to lead to exposure misclassification, which may underestimate the effects of air pollution [44]. Another limitation was our inability to explore the independent effect of PM_2.5_ because data on other air pollutants was not available in this study. Therefore, our results should be cautiously interpreted, and future studies are needed to explore the independent effect of PM_2.5_ on COPD. Third, this analysis did not take into account several potential confounding factors, e.g., socioeconomic status and daily activities. Finally, we used ambient PM_2.5_ levels on the day of hospital visits (outpatient and inpatient visits) as individual exposure. This strategy is consistent with previous studies [4, 5, 22, 25]. However, inpatients would be in hospital, being less exposed to the ambient PM_2.5_ concentrations. This exposure measurement error would tend to bias the effect estimates downward [44].

## Conclusions

In conclusion, this study suggests that short-term exposure to ambient PM_2.5_ may account for increased daily hospital visits for COPD exacerbations in Beijing, China. Among COPD patients, elderly people and females were more sensitive to the air pollution effects. More attention should be paid to these subpopulations.

## Additional file

Additional file 1: **Figure S1.** The U.S. Embassy in Beijing is shown at the center of the red circle, the radius of which is 40 km. **Figure S2**. A scatter plot on PM_2.5_ concentrations and counts of outpatient and inpatient visits. **TableS1**. Percentage changes with 95% CIs in outpatient and inpatient visits for chronic obstructive pulmonary disease (COPD) associated with per 10 μg/m^3 ^increase in fine particulate matter (PM_2.5_) concentration for different lag structures. (PDF 195 kb)
