# Supplementary material for: Short-term effects of ambient fine particulate matter pollution on hospital visits for chronic obstructive pulmonary disease in Beijing, China
Source: Environ Health. 2018 Feb 27;17:21. doi: 10.1186/s12940-018-0369-y (PMC6389038; doi:10.1186/s12940-018-0369-y)
Supplement: Supplementary file 1 — Figure S1. The U.S. Embassy in Beijing is shown at the center of the red circle, the radius of which is 40 km. Figure S2. A scatter plot on PM2.5 concentrations and counts of outpatient and inpatient visits. TableS1. Percentage changes with 95% CIs in outpatient and inpatient visits for chronic obstructive pulmonary disease (COPD) associated with per 10 μg/m3 increase in fine particulate matter (PM2.5) concentration for different lag structures. (PDF 195 kb) [file 12940_2018_369_MOESM1_ESM.pdf]

Short-term effects of ambient fine particulate matter pollution on hospital visits for chronic obstructive pulmonary disease in Beijing, China

Yaohua Tian [1], Xiao Xiang [1], Juan Juan [1], Jing Song [1], Yaying Cao [1], Chao Huang [1], Man Li [1], Yonghua Hu [1,*]

[1] Department of Epidemiology and Biostatistics, School of Public Health, Peking University, No.38 Xueyuan Road, 100191 Beijing, China

Yaohua Tian and Xiao Xiang contributed equally.

[*] **Corresponding author:** Yonghua Hu Prof., M.D, department of Epidemiology and Biostatistics, School of Public Health, Peking University, No.38 Xueyuan Road, 100191 Beijing, China, Tel.: +86-135-0136-1139, Fax: +86-10-82801189, E-mail: yhhu@bjmu.edu.cn

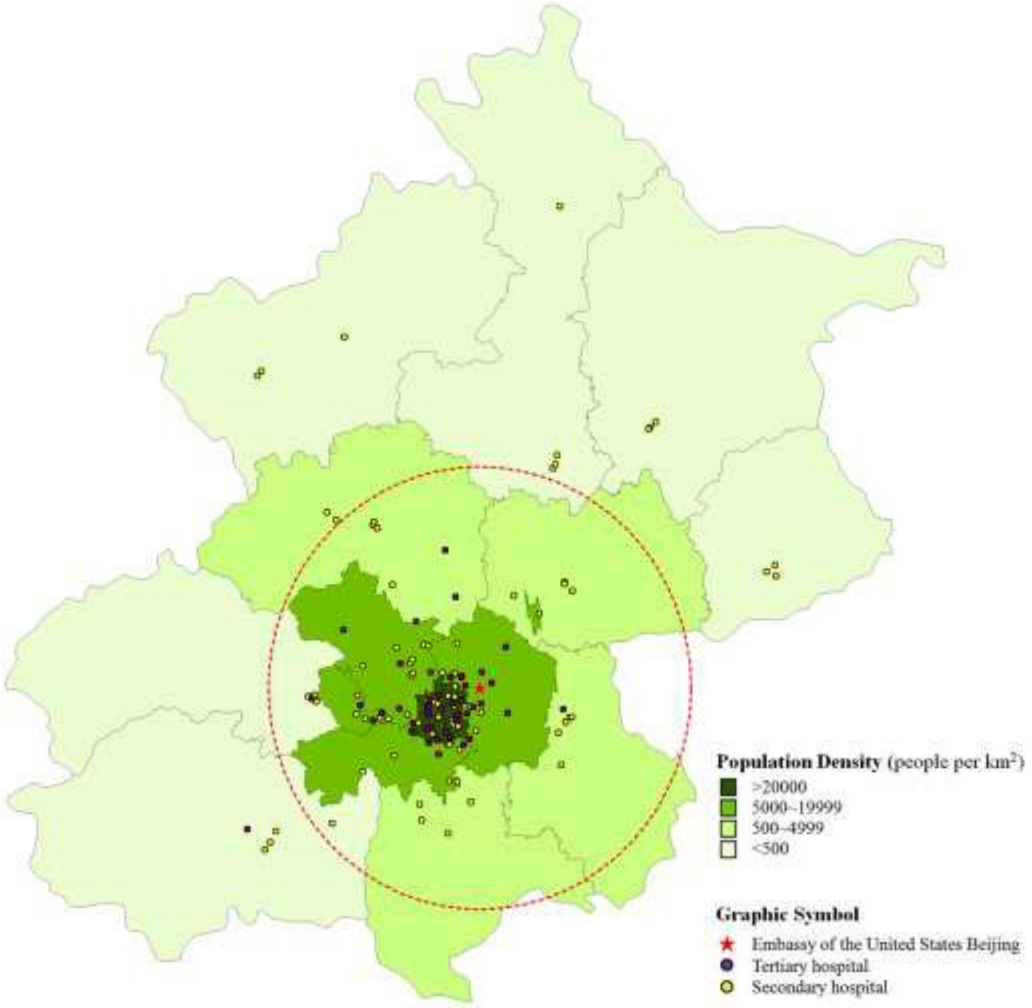

Figure S1. The U.S. Embassy in Beijing is shown at the center of the red circle, the radius of which is 40 km.

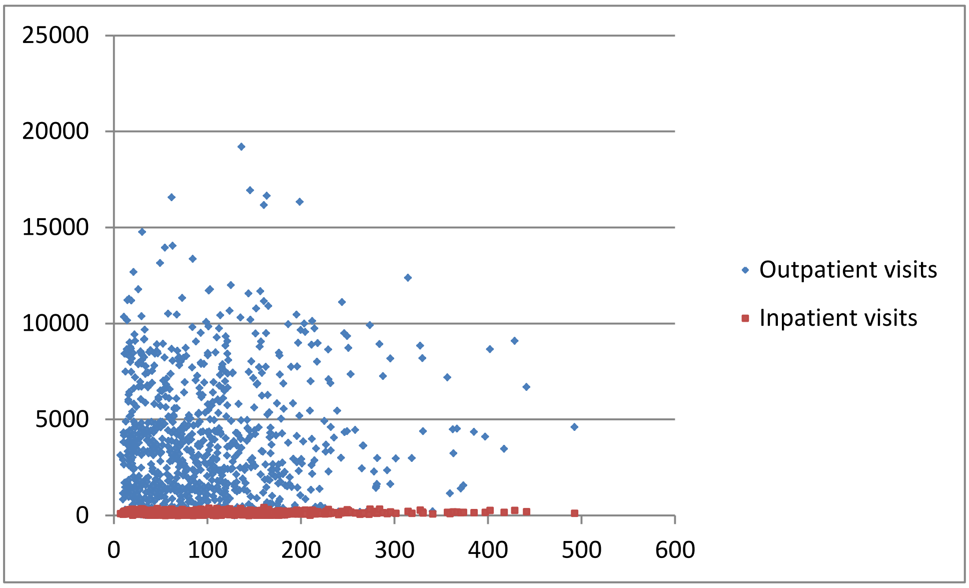

Figure S2. A scatter plot on $PM_{2.5}$ concentrations and counts of outpatient and inpatient visits.

Table S1. Percentage changes with 95% CIs in outpatient and inpatient visits for chronic obstructive pulmonary disease (COPD) associated with per 10 ug/m^3 increase in fine particulate matter ($PM_{2.5}$) concentration for different lag structures.

Hospital service	Lag days	Percentage change	95% CI	*P*
Outpatient visits	Lag 0 days	0.26	0.24-0.28	<2e-16
	Lag 1 days	0.02	0.01-0.04	0.00485
	Lag 2 days	0.10	0.08-0.11	<2e-16
	Lag 3 days	0.17	0.16-0.18	<2e-16
	Lag 0-1 days	0.18	0.16-0.20	<2e-16
	Lag 0-2 days	0.20	0.18-0.22	<2e-16
	Lag 0-3 days	0.26	0.24-0.29	<2e-16
Inpatient visits	Lag 0 days	0.65	0.56-0.73	<2e-16
	Lag 1 days	0.26	0.18-0.34	4.46e-11
	Lag 2 days	0.14	0.07-0.21	0.000119
	Lag 3 days	0.02	-0.05-0.09	0.527
	Lag 0-1 days	0.60	0.50-0.70	<2e-16
	Lag 0-2 days	0.55	0.44-0.65	<2e-16
	Lag 0-3 days	0.47	0.36-0.58	<2e-16
